# Effects of feeding naturally contaminated deoxynivalenol diets to sows during late gestation and lactation in a high-yield specific pathogen-free herd

**DOI:** 10.1186/s40813-018-0102-9

**Published:** 2018-11-01

**Authors:** Amin Sayyari, Tore Framstad, Anette Kristine Krogenæs, Tore Sivertsen

**Affiliations:** Department of Production Animal Clinical Sciences, Faculty of Veterinary Medicine, Norwegian University of Life Sciences, P.O. Box 369, Sentrum, 0102 Oslo, Norway

**Keywords:** Deoxynivalenol, *Fusarium*, Sow, Production, Reproduction, Performance

## Abstract

**Background:**

The most prevalent *Fusarium* mycotoxin in grains is deoxynivalenol (DON). Contamination of swine feed with DON can result in reduced consumption and poor growth performance. Gestating and lactating sows need sufficient feed intake for fetus development during late gestation and milk production and body maintenance during lactation. Therefore, there is considerable concern in modern piglet production about the effects of DON contamination in sow feed. Most previous studies in sows have been done under experimental conditions, with DON levels ≥2.8 mg/kg feed. The aim of the current field trial was to investigate the effects of feeding grains that are naturally contaminated with more realistic levels of DON on sows during late gestation and lactation.

**Methods:**

In a commercial, high-yield specific pathogen-free piglet production unit, 45 Norwegian Landrace × Yorkshire sows were fed three diets from 93 ± 1 days of gestation until weaning of the piglets, and average daily feed intake (ADFI), body weight (BW), production and reproduction performance, as well as sow blood parameters were recorded. Diets were made from naturally contaminated oats, with three concentration levels: 1) control (DON < 0.2 mg/kg), 2) DON level 1 (1.4 mg DON/kg), and 3) DON level 2 (1.7 mg DON/kg).

**Results:**

Sows that were fed DON level 1 and 2 diets showed a 4–10% reduction in feed consumption during lactation, compared with sows in the control group. However, the DON-contaminated diets did not significantly affect sow BW or backfat thickness. Similarly, there were neither effects on production or reproduction performance, nor on blood parameters in the sows. The effects on skin temperature were variable.

**Conclusion:**

Naturally contaminated diets with realistic, moderately increased DON levels, fed during late gestation and lactation in a modern high-yield piglet production farm, had limited effects on sow health and production.

**Electronic supplementary material:**

The online version of this article (10.1186/s40813-018-0102-9) contains supplementary material, which is available to authorized users.

## Background

For many years, deoxynivalenol (DON) has been known as one of the most important trichothecene mycotoxins that commonly contaminates grain. Swine are particularly sensitive to DON exposure because of delayed excretion and poor detoxification [1]. Dose-related feed refusal, reduction in feed intake, and reduced weight gain of growing pigs have been associated with DON-contaminated diets [2, 3]. However, only a limited number of studies have researched the effects of feeding diets that are naturally contaminated with DON to sows during late gestation and lactation. Some of these studies did not show a significant effect of DON levels from 2.8 to 6.2 mg/kg feed; moreover, these levels did not affect feed intake, body weight (BW) change, or litter performance [4, 5]. Chavez [6], on the other hand, reported significantly higher BW loss in the sows that were fed diets contaminated with 3.3 mg DON/kg, while feed intake and litter performance were not affected. Diaz-Llano and Smith [7] and Diaz-Llano et al. [8] also reported significantly lower feed intake and higher BW loss during lactation when fed diets that contained 5.5 mg DON/kg [7] or 3.6 mg DON/kg [8], compared to the control groups. However, litter performance was not significantly affected [7]. Jakovac-Strajn et al. [9] reported lower feed intake and litter weight gain and longer duration of farrowing in sows that received diets containing 5 mg DON/kg, compared to control sows, while sow BW was not affected. Feeding lactating sows with diets containing up to 3 mg DON/kg decreased feed intake, decreased BW, and increased backfat loss, although litter performance and sow reproductive performance were not affected [10].

Most of these studies were conducted under controlled, experimental conditions, and with levels of DON contamination ≥2.8 mg/kg. However, in Norway, some farmers and veterinarians have reported suspected cases of negative DON-related effects on sow health and litter performance after intake of feed with more moderate DON levels [11]. In the last decade, average litter size and lactation performance of sows in Norway have increased substantially [12]. It is, therefore, a relevant question whether high-yielding sows in modern pig production may be more sensitive to moderate levels of DON than results from previous studies should indicate. Accordingly, we investigated the effects of diets that were naturally contaminated with realistic, moderately increased levels of DON on sows in late gestation and during lactation. We studied the effects on sow feed intake, BW development, litter gain, and reproduction performance in a field trial, conducted under practical conditions in a modern, high-yield piglet production unit. In addition, we studied the effect of different levels of DON contamination on hematology and biochemistry parameters, skin temperature, litter size, number of stillborn piglets, and production results in the subsequent litter.

## Methods

### Animals, housing, and management

The trial was done in a commercial specific pathogen-free (SPF) high-yield piglet production unit in southeastern Norway. Table 1 shows the key performance indicators of the farm in 2015. Norwegian SPF herds are free from specific swine diseases and parasites such as *Sarcoptes scabiei*, *Brachyspira hyodysenteriae*, *Brachyspira pilosicoli*, *Actinobacillus pleuropneumonia*, *Mycoplasma hyopneumoniae*, toxin-producing *Pasteurella multocida*, *Lawsonia intracellularis*, *Salmonella* sp. and *Ascaris suum*.

**Table 1:** Key performance indicators of the piglet farm in 2015

Item	Value
Total pigs weaned	8898
Farrowing rate, %	83.6
Pigs born alive / Litter	14.1
Stillborn pigs / Litter	1.5
Pigs weaned / Litter	12.3
Average age at weaning, *days*	33
Average individual weaning weight, *kg*	11.6
Pre-weaning mortality, *%*	13.1
Average gestation length, *days*	115
Pigs weaned / sow /year	27.2
Litter / sow / year	2.22
Weaning to 1st service interval, *days*	5.3
Non-productive days / Litter	15

This trial was performed in a farrowing group of 47 Norwegian Landrace × Yorkshire sows for 53 days from December 2015 to February 2016. All sows were kept individually and without fixation in standard farrowing pens (7.0 m^2^) with a piglet creep area (1.3 m^2^), from approximately 3 weeks before expected farrowing until weaning. Each pen had a solid concrete floor, except for a slatted draining floor at one end of the pen (2.3 m^2^). The sows were distributed based on parity numbers, to ensure even distribution of parity within groups.

The sows were further divided into three feeding groups: (1) control (*n =* 16), (2) DON level 1 (*n =* 15), and (3) DON level 2 (*n =* 16). They were fed twice daily during gestation until 1 week postpartum, when feeding frequency was increased to three times daily, and four times daily from 2 weeks postpartum to weaning. The sows were offered a restricted amount of feed during late gestation, with a maximal allowance of 4 kg/day to each sow in this period. After farrowing, the amount of feed offered to each sow was gradually increased and the feeding automates adjusted to meet the requirements for a modified ad libitum feeding strategy. The amount of feed offered to each sow by the automated feeding system was adjusted continuously; in such a way that a small amount of feed was left over in the trough before the next feeding, as a confirmation that the sows had access to feed ad libitum. After weaning, the sows were removed from the farrowing unit and fed uncontaminated diets. All sows were offered 0.2 kg of hay daily. Both sows and piglets had access to water ad libitum. Farrowing occurred naturally, although under constant staff surveillance. Commercial husbandry procedures were performed within 48 h after birth, including teeth grinding and a 200-mg oral iron supplement (1.5 mL plus iron pasta, Felleskjøpet, Norway). Thereafter, a creep feed mixed with iron-fortified peat was offered to the piglets on the concrete floor of the piglet creep area until 4 days before weaning. Between 5 and 10 days after farrowing, a local veterinarian performed surgical castration using local anesthesia and analgesia. Necessary cross-fostering of piglets was performed within or across treatments within 48 h after farrowing and recorded. All litters were weaned on the same day, and average lactation length was 33.3 days (range 29–38 days).

Two sows (one from the control group and one from the DON level 2 group) were removed from the trial. The omitted sow from the control group died during the lactation period due to a penetrating gastric ulcer, and the other one was excluded from the study because the sow did not eat the experimental diet at all, and consequently received other feed. The final study therefore included 45 sows; 15 in each feeding group.

### Origin of the naturally contaminated oats

The DON-contaminated oats that were used to produce the experimental diets in the present study were harvested in southern Sweden in 2013 and provided by Lantmännen, Sweden. The experimental diets in this study contained oats from the same DON-contaminated batch that was used in our recent study on growing pigs [13]. This batch of oats was analyzed for a wide range of mycotoxins, using a semi-quantitative multi-toxin screening method, at the Centre for Analytical Chemistry at IFA Tulln, Austria (Additional file 1: Table S1) [14].

### Preparation of the experimental diets

From arrival in the farrowing unit, the sows were fed pelleted feed with different levels of naturally DON-contaminated oats (Table 2). The experimental diets were produced by a standard pelleting process by Felleskjøpet Agri, Lillestrøm, Norway (Production site: Trondheim, Norway), and were based on the formula of a commercial lactation diet (FORMAT, Felleskjøpet Fôrutvikling, Trondheim, Norway). The three diets were formulated to provide the following treatments: (1) control-diet (DON < 0.2 mg/kg), (2) DON-contaminated diet level 1 (DON = 1.4 mg/kg), and (3) DON-contaminated diet level 2 (DON = 1.7 mg/kg). We initially planned to run the trial with DON levels of approximately 0.5 mg/kg and 1 mg/ kg, in the two DON-contaminated diets. The different dose levels were sought by blending the naturally contaminated oats with very low-contaminated oats, which were harvested in Norway in 2014. However, due to an unexplained factor in the feed production at the factory, the final contaminated diets contained higher and more similar levels of DON than originally planned.

**Table 2:** Experimental diet composition

	Experimental Diets			
Ingredients (as-fed basis)	Control	DON- Level 1	DON- Level 2	Unit
Barley, uncontaminated	18.8	18.8	18.7	%
Wheat, uncontaminated	25	25	25	%
Oats, uncontaminated	20	13.6	5.5	%
Oats, contaminated	0	6.4	14.5	%
Soybean meal	12	12	12	%
Rapeseed cake Mestilla	5	5	5	%
Corn gluten meal	1.04	1.04	1.04	%
Corn grits	2	2	2	%
Wheat bran meal	2	2	2	%
Molasses	2	2	2	%
Cossettes	3	3	3	%
Fish meal	1	1	1	%
Fish protein concentrate (by Scanbio)	3	3	3	%
Animal fat	3.05	3.05	3.05	%
Calcium carbonate (Limestone)	1.26	1.26	1.26	%
Monocalcium phosphate	0.6	0.6	0.6	%
Sodium bicarbonate	0.03	0.03	0.03	%
Salt	0.43	0.43	0.43	%
Sel-plex 1000	0.015	0.015	0.015	%
Selen premix	0.04	0.04	0.04	%
Mikromin. Swine	0.16	0.16	0.16	%
Vit.premix	0.06	0.06	0.06	%
Vitam-ADKB	0.07	0.07	0.07	%
L-lysin	0.26	0.26	0.26	%
DL-methionin	0.04	0.04	0.04	%
L-treonin	0.10	0.10	0.10	%
L-valin	0.01	0.01	0.01	%
Tryptophan	0.01	0.01	0.01	%
Betain	0.01	0.01	0.01	%
Digestrarom 1310	0.02	0.02	0.02	%
Ronozyme hiphos (0-50 g/t)	0.01	0.01	0.01	%
Vitamin E 50%	0.03	0.03	0.03	%
Biotin 10%	0.002	0.002	0.002	%
Calculated composition (as-fed basis)				
Protein	16.4	16.4	16.4	%
Fat SOX	5.58	5.59	5.59	%
Fat HCL	6.2	6.2	6.21	%
Water	12.3	12.3	12.3	%
Starch	34.8	34.8	34.8	%
Lysine	0.99	0.99	0.99	%
Methionine	0.3	0.3	0.3	%
Methionine + cysteine	0.62	0.62	0.62	%
Threonine	0.69	0.69	0.69	%
Tryptophan	0.2	0.2	0.2	%
Ca	0.84	0.84	0.84	%
P	0.53	0.53	0.53	%
Na	0.23	0.23	0.23	%
NE	10.2	10.2	10.2	(MJ/kg)
Deoxynivalenol	< 0.2	1.4	1.7	(mg/kg)
3-ac-DON	< 0.01	0.14	0.17	(mg/kg)
15-ac-DON	< 0.05	≤ 0.05	< 0.05	(mg/kg)
DON-3-Glc	< 0.03	0.32	0.36	(mg/kg)

### Analysis of mycotoxins in experimental diets

The experimental diets were analyzed at the Norwegian Veterinary Institute for DON, DON-3-Glc, 3-ac-DON, and 15-ac-DON with a previously validated liquid chromatography-high resolution mass spectrometry method [15]. The limits of detection (LOD) in the feed matrix were 14 μg/kg for DON, 26 μg/kg for DON-3-Glc, 5.9 μg/kg for 3-ac-DON, and 52 μg/kg for 15-ac-DON. Increased DON concentrations were the dominant mycotoxin contamination recorded in the experimental feeds in the current study, and the other commonly occurring mycotoxins in Norwegian cereal grain were below the LOD or detected at insignificant levels. The hay provided to the sows was not analyzed for mycotoxin content. However, all the hay was of good quality, with no sign of fungal contamination.

### Clinical examination and skin temperature

The animals were monitored for clinical diseases and disorders during the experiment. We recorded all diseases and treatments in both sows and piglets. Skin temperatures from the sow’s ear were recorded upon arrival in the farrowing unit, 14 days after arrival, within 10–36 h after farrowing, on day 7 and 21 during the lactation period and at weaning, using a thermal imaging camera (FLIR i7, FLIR System, Inc., Wilsonville, OR, USA).

### Feed intake and growth performance

In each farrowing pen, a plastic dispenser showed the amount of feed intake at each feeding. In this study, daily feed consumption refers to feed disappearance from the dispenser and was not adjusted for feed spillage or consumption by piglets.

Upon arrival at the farrowing unit, all sows were weighed individually, using a digital scale (EC2000, TEO teknikk AS, Nærbø, Norway) with a dimension of 1 × 2 m and an accuracy of 500 g. They were weighed again after 14 days, in late gestation period. Within 12–36 h after parturition, the sows and litters were weighed. Litter weight was measured using a bucket placed on a digital walk-on scale (Slim-Line small animal weight scale, Eickemeyer Medizintechnik für Tierärzte, KG, Tuttlingen, Germany) with a dimension of 90 × 55 cm and an accuracy of 100 g. Sows and litters were also weighed on days 7 and 21 of lactation and at weaning.

### Backfat measurement and body condition score

Backfat measurements were done by a trained technician from Norsvin (Norwegian Pig Breeding Association). Due to practical limitation, backfat measurement was performed only twice: postpartum (1–7 days postpartum, as all sows did not farrow on the same day) and at weaning. Backfat was measured using RKU 10, a veterinary B mode ultrasound scanner (KAI XIN, Jiangsu, China), over the second-to-last rib of the sows. The measurements are presented in *mm*.

Body condition scores (BCS) were performed on all sows, using the scale developed by Animalia and Norsvin [16]. All sows were given a BCS ranging from 1 to 5, with half-point gradations: 1 is emaciated, 2 is thin, 3 is good condition, 4 is overweight, and 5 is obese. BCS was given upon arrival in the farrowing unit, after 14 days, 12–36 h after parturition, on days 7 and 21 during lactation, and at weaning.

### Litter and reproductive performance

On the day of parturition, we recorded gestation length and duration of farrowing (time between the first and the last piglet born). The number of total born, born alive, and stillborn piglets were recorded for each sow. Within 12–36 h after parturition, each litter of live piglets was weighed. Stillborn piglets in each litter were registered. Litters were also weighed and litter size was recorded on days 7 and 21 during the lactation period and at weaning. After weaning, we recorded the fate of all sows used in the trial, and the number of sows kept for further production that returned to heat after 5 days. Non-pregnant sows were culled and urogenital organs were examined for type of fertility disturbances. Finally, we recorded the number of conceptions at first service, and the number of live piglets born to the sows in the subsequent parity.

### Clinical chemistry and hematology

Blood samples from the sows were taken from the milk vein (*v. subcutanea abdominis*) [16], using 9-mL heparin tubes for chemistry and 5-mL ethylenediaminetetraacetic acid (EDTA) tubes for hematology. Blood samples were taken upon arrival to the farrowing unit, 10 days after arrival, within 12–36 h after parturition, and at weaning. The EDTA tubes were kept refrigerated and delivered to the Central Laboratory at the Norwegian University of Life Sciences (NMBU), Faculty of Veterinary Medicine (Oslo, Norway), on the same day or in the next morning. Samples for plasma chemistry and biochemistry were prepared by centrifugation at 1500×g for 10 min at room temperature (approximately 20 °C), stored in 2-mL cryogenic vials (Nalgene, Nalge Company, Rochester, NY, USA), and delivered to the Central Laboratory.

For hematological analyses, we used ADVIA® 2120 Hematology System ADVIA® Multispecies software (Siemens Healthcare Diagnostics, Siemens AG, Erlangen, Germany), with the settings for swine species. The clinical biochemical analysis was performed using ADVIA 1800® Clinical Chemistry System (Siemens Healthcare Diagnostics, Siemens AG, Germany), and serum protein electrophoresis was performed on Sebia CapillarysTM 2 (Sebia, Norcross, GA, USA). The biochemical analyses included aspartate aminotransferase (AST), alkaline phosphatase (ALP), gamma-glutamyl transpeptidase (GGT), glutamate dehydrogenase (GLDH), creatine kinase (CK), C-reactive protein (CRP), total serum protein, total serum globulins, urea, creatinine, total bilirubin, cholesterol, glucose, inorganic phosphate, and calcium.

Blood samples from the sows and selected piglets were also collected for analysis of DON uptake, metabolism and vertical transmission. These results will be published in a separate paper.

### Statistical analysis

After the trial, the analyses of DON concentrations in plasma indicated a mistake in the feeding of one of the sows in the DON level 2 group. The plasma concentrations of DON and its metabolites in this sow were below the LOD at 5 of 8 measurements. This sow was therefore excluded from all statistical analyses. Thus, the final dataset included 44 sows (15 in the control, 15 in the DON level 1, and 14 in the DON level 2), where 11 were first parity sows (4 in the control, 4 in the DON level 1, and 3 in the DON level 2), 12 were second parity sows (4 in each treatment), and 21 sows ranged from parity 3–6 (7 in each treatment).

All statistical analysis in this study was performed in JMP®, Version 10 (SAS Institute Inc., Cary, NC, USA). The level of significance was set to 0.05 in all models, and results with *p*-values between 0.05 and 0.1 were considered trends. If not otherwise specified, all results are expressed as means ± standard deviation (SD). The data were considered as a completely randomized block design with three treatments in 15 blocks. Each sow and its litter were considered as a random effect and represented an experimental unit for tested variables. The experimental diets were the independent variables. Growth performance parameters, skin temperature, and hematological and biochemical parameters were defined as dependent variables. The normality of distribution was controlled by residual and predicted values plot, normal-percentile plots, and Shapiro-Wilk test. If the *p*-value in the Shapiro-Wilk test was over 0.05, data were considered normally distributed. Data that were not normally distributed were transformed or analyzed by non-parametric models, such as Wilcoxon rank-sum and Kruskal-Wallis tests. The same tests were performed when the data failed the assumptions of the analysis of variance (ANOVA). If the data generated from the application of ANOVA were significantly different, the post hoc, Tukey-Kramer honest significant difference (HSD) test was used for multiple comparisons and distinction of significant differences (*p* <  0.05). The Steel-Dwass test was used for multiple comparisons of the data generated from the application of Kruskal-Wallis test (*p* <  0.05).

Statistical differences between the treatment groups (control diet, DON level 1, and DON level 2 diets) for changes in sow weight, feed consumption, weight loss, litter weight, skin temperature, hematological and biochemical data, total backfat, and total litter gain were analyzed using a mixed effects model, with treatment, parity, and their interaction as fixed effects and individuals nested to treatments as a random effect. When appropriate, number of piglets born alive, number of weaned piglets, and lactation length were included in the models as covariates. Covariates and interactions that were not statistically significant were removed from the models by backward elimination.

Log-linear regression with normal distribution was used for data such as total number born, number born alive, and number of liveborn piglets in subsequent parity, with a Poisson distribution for the number of weaned piglets and negative binomial distribution for gestation length and duration of farrowing.

## Results

We conducted a post-mortem investigation of the sow in the control group that died during lactation, and encountered penetrating gastric ulcers. There were no DON-related health issues, such as vomiting, diarrhea, or other pathological conditions, in any of the other sows during the trial.

### Effects of experimental diets on feed consumption

During late gestation, ADFI of the sows were 3.76 ± 0.21*,* 3.36 ± 0.30 and 3.66 ± 0.33 *kg* in the control, DON level 1 and DON level 2 groups, respectively (Table 3). During lactation, sows fed the DON level 2 diet had significantly lower ADFI (6.12 ± 0.41 *kg*) (*p* <  0.01; Table 3) than the control group (6.76 ± 0.56 *kg*; Table 2). In the same period, sows receiving the DON level 2 diet had a non-significant lower ADFI than sows in the DON level 1 group (6.52 ± 1.20 *kg*) (*p* = 0.33) while there was no significant difference between the DON level 1 and the control groups (Table 3). The interaction between parity and treatment (DON) had a statistically significant effect on feed consumption during lactation, *F* (10, 25) = 5.90, *p* = 0.0002.

**Table 3:** Effect of DON on sow growth performance and skin temperature

Item	Diets						Comparison *p*-values		
	1: Control (*n*=15)		2: DON-Level 1 *(n*=15)		3: DON- Level 2 (*n*=14)		1 vs. 2	1 vs. 3	2 vs. 3
	Mean	SD	Mean	SD	Mean	SD			
Average Daily feed intake (ADFI), *kg/day*									
During late gestation	3.76	0.21	3.36	0.30	3.66	0.33	–	–	–
During lactation ^a^	6.76	0.56	6.52	1.20	6.12	0.41	0.83	0.01	0.33
Sow weight, *kg*									
Arrival farrowing unit	260	37	265	30	262	24	0.89	0.97	0.96
Day 14 after arrival ^b^	296	38	301	33	303	25	0.93	0.95	0.99
12–36 h after farrowing	268	37	280	38	277	26	0.45	0.84	0.83
Day 7 after farrowing	260	36	273	38	265	26	0.44	0.99	0.54
Day 21 after farrowing	254	39	262	39	257	33	0.30	0.90	0.17
Weaning	251	42	258	40	255	34	0.83	0.99	0.81
Weight gain in late gestation ^c^	36.5	5.8	36.7	7.2	40.6	7.6	0.99	0.29	0.29
Weight loss wk. 1–2 after farrowing	7.8	3.4	7.2	6.3	11.6	9.2	0.98	0.26	0.19
Weight loss wk. 2–3 after farrowing	6.3	12.0	10.8	6.4	8.57	12.6	0.47	0.91	0.73
Weight loss in lactation	17.2	17.8	22.1	14.9	22.3	18.9	0.72	0.71	0.99
Backfat rib, *mm*									
1–7 days after farrowing	14.6	5.9	16.9	5.9	18.3	5.3	0.33	0.19	0.79
weaning	12.1	5.0	12.7	3.6	13.5	6.7	0.53	0.95	0.99
Total backfat loss rib	2.5	3.8	4.3	4.6	5.5	4.3	0.51	0.16	0.71
Skin temperature, *°C*									
Arrival farrowing unit	32.2	2.0	31.8	1.7	31.8	2.2	0.51	0.94	0.94
Day 14 after arrival	34.0	0.8	33.9	1.2	33.9	0.8	0.97	0.65	0.97
12–36 h after farrowing	35.2	1.2	35.0	1.3	33.8	1.5	0.67	0.02	0.24
Day 7 after farrowing	34.2	1.5	34.5	1.0	34.1	1.0	0.93	0.75	0.46
Day 21 after farrowing	35.3	1.1	35.3	1.2	35.5	0.8	0.99	0.97	0.98
Weaning	35.9	1.0	35.9	0.6	35.1	0.8	0.94	0.06	0.03

^a^One sow in control was excluded from the final analysis of this variable

^b^Two sows in control were excluded from the final analysis of this variable

^c^Two sows in control and one sow in DON-level 2 were excluded from the final analysis of this variable

### Effects of experimental diets on sow and litter performance and on subsequent reproduction

No statistically significant differences were found in sow weights at the start of the trial, 14 days after arrival, within 12–36 h after farrowing, on day 7, day 21, or at weaning (Table 3). The sows in the two DON groups had higher average weight losses during lactation compared to sows in the control group (Table 3). However, due to large individual variation, the group differences were not statistically significant. Similarly, the interaction between parity and treatment (DON) did not have a statistically significant effect on sow weight, *F* (10, 26) *=* 1.35*, p =* 0.26.

There were also no statistically significant differences between the treatment groups in backfat depth measured at 1–7 days postpartum and at the time of weaning. (*p* > 0.05). However, sows in the DON level 2 (5.5 ± 4.3 *mm*) and DON level 1 (4.3 ± 4.6 *mm*) groups had a higher average backfat loss at the end of the trial than the control group (2.5 ± 3.8 *mm*). Again, due to large individual variation, the group differences were not statistically significant (Table 3). Likewise, there were no statistically significant differences in BCS. The interaction between parity and treatment (DON) did not have a statistically significant effect on total backfat loss, *F* (10, 26) = 1.07, *p* = 0.41.

No statistically significant differences were found in gestation length or duration of farrowing, although, the mean duration of farrowing was 69 min longer in the DON level 2 group than the control group. The total number born, born alive, and weaned piglets did not differ between the groups (Table 4).

**Table 4:** Effect of DON on sow reproductive performance

Item	Diets						*p*-value
	Control		DON- Level 1		DON- Level 2		
	Mean	SD	Mean	SD	Mean	SD	
Reproduction							
Gestation length, *days*	114.53	1.13	115.13	1.06	115.07	1.68	0.99
Duration of farrowing, *minutes* ^a^	448	190	454	138	517	208	0.54
Time interval between birth of each piglet, *minutes* ^a^	33	25	34	22	36	18	0.74
Total born, *n*	16.9	5.1	15.9	4.0	15.4	4.7	0.67
Born alive, *n*	15.3	4.5	14.7	3.3	13.6	4.2	0.56
Weaned, *n*	11.6	2.1	12.9	0.9	11.0	2.1	0.34
Sows returning to heat at 5 days after weaning, *n* ^b^	13	–	14	–	11	–	–
Conceptions at first subsequent service, *n* ^b^	10	–	12	–	9	–	–
Born alive subsequent litter, *n*	16.9	3.0	15.3	4.2	14.7	2.6	0.33

^a^One sow in DON-level 2 was excluded from the final analysis of this variable

^b^Total number of sows returning to heat and total number of conceptions

Litter weight at birth (12–36 h after parturition), day 7 and day 21 after parturition, and at weaning did not differ significantly between the treatment groups (*p* > 0.05). On average, the sows that received the DON level 2 diet had lower litter gain, but the difference was not statistically significant (Table 5).

**Table 5:** Effect of DON on sow productive performance

Item	Diets						Comparison *P*-values		
	1: Control		2: DON- Level 1		3: DON- Level 2		1 vs. 2	1 vs. 3	2 vs. 3
	Mean	SD	Mean	SD	Mean	SD			
Production									
Litter weight at birth, *kg*	21.1	3.6	20.5	2.7	21.6	4.5	0.37	0.74	0.14
Litter weight day 7, *kg*	34.7	7.0	35.3	3.3	32.9	8.2	0.94	0.58	0.52
Litter weight day 21, *kg*	83.3	14.7	88.1	10.7	81.4	16.9	0.85	0.99	0.81
Litter weaning weight, *kg*	138	26	142	12	128	27	0.24	0.93	0.41

The day after removal from the farrowing unit, one sow in the DON level 1 group died; also from a penetrating gastric ulcer. Four sows were sorted out for slaughter; due to age (2 sows), damaged teats (1) and lameness (1). Of the remaining sows, one in the control group did not return to heat in 5 days, and was sorted out on that basis. All the other sows returned to heat at 5 days post-weaning. The number of conceptions at first service was similar in all treatment groups, and there were no significant differences in the average number of liveborn piglets per sow in the subsequent litter (*p* > 0.05; Table 4). The results from pathological examination of urogenital organs of non-pregnant sows revealed endometritis due to infection with *Escherichia coli* or *Staphylococcus hyicus* in 3 sows from the control, 2 sows from DON level 1 and 2 sows from DON level 2.

Regardless of treatment and parity, a negative correlation was observed between the sows’ total feed consumption and weight loss during lactation (*R* = – 0.39, *p* < 0.01). Several interactions were positively correlated (Table 6): total feed consumption during lactation and litter weaning weight (*R* = 0.28, *p* = 0.06); sows’ total weight loss during lactation and litter weaning weight (*R* = 0.44, *p* < 0.01); and sows’ total weight loss during lactation and backfat loss (*R* = 0.47, *p* < 0.01).

**Table 6:** Correlations between feed consumption, weight loss, litter weaning weight, and backfat loss during lactation

Variable	1	2	3	4
1. Feed consumption, *kg*	1.00			
2. Weight loss sow, *kg*	–0.39 **	1.00		
3. Litter weaning weight, *kg*	0.28	0.44**	1.00	
4. Total backfat loss sow, *mm*	–0.15	0.47**	0.16	1.00
Mean	229.1	20.5	136.2	4.1
SD	32.5	17.0	22.8	4.3
Range	(138.0-278.1)	((−13.0)-49.0)	(82.7-175.8)	((−5.0)-16)

**p < 0.01

### Effects of experimental diets on skin temperature

There were no significant differences in skin temperature between sows fed experimental diets and the control, throughout most of the experiment. However, sows in the DON level 2 group had a significantly lower skin temperature (33.8 ± 1.5 *°C*; *p* < 0.05) than sows in the control group (35.2 ± 1.2 *°C*) immediately after farrowing. Sows in the DON level 2 group also had a significantly lower skin temperature (35.1 ± 0.8 *°C*; *p* < 0.05) than sows in the DON level 1 group (35.9 ± 0.6 *°C*) at weaning. Additionally, they had a tendency (*p* = 0.06) to have a lower temperature than the control group (35.9 ± 1.0 *°C*) at weaning (Table 3).

### Effect of experimental diets on hematological and biochemical parameters

Hematological parameters, including red and white blood cell counts, hematocrit, hemoglobin, and platelets did not change with DON level or time.

No differences were observed between the groups, throughout the experiment, in any of the measured biochemical parameters.

## Discussion

### Feed consumption

In the present study, the sows fed contaminated diets had a lower feed consumption rate during lactation than the control group did. This finding is in line with those of Diaz-Llano and Smith [7], Diaz-Llano et al. [8], Jakovac-Strajn et al. [9], and Herkelman et al. [10], who found reduced feed intake in sows fed diets with higher DON levels (3 to 5.5 mg/kg). Diaz-Llano et al. [8] observed a reduction in feed intake only during lactation. Our results contrast those of Chavez [6], who reported that feed intake of gilts was not affected by diets containing up to 3.3 mg/DON kg. In that study, the feeding was restricted in late gestation (2.3 kg/day) and ad libitum during lactation. The results of the present study also diverge from those of Friend et al. [4], Diaz-Llano and Smith [17], and Gutzwiller [5], who reported that restricted feeding of gilts, with diets containing 2.8 to 6.2 mg DON/kg, did not reduce feed consumption during gestation or lactation. Given that the feeding was restricted (up to 4 kg/day) in late gestation in the present study, the differences in ADFI between sow groups in this period should be treated with considerable caution. Thus, we have not analyzed these differences statistically. The ADFI in late gestation was strongly influenced by the feeding method, as the sows were offered a standard amount of feed, according to the routines of the farmer. Therefore, the differences recorded in feed intake in this period do probably not reflect the effect of DON content in the feed.

When comparing results from different feeding experiments, one should consider that the recorded effects of DON-contaminated feed in pigs may be influenced by different feeding strategies [18]. Some studies reported in sows have used restricted feeding in both gestation and lactation, while the other studies have used a combination of restricted feeding during late gestation and ad libitum feeding during lactation. In the present study, we have used a restricted feeding during late gestation and a modified ad libitum feeding strategy, using a semi-automatic feeder during lactation.

Furthermore, one cannot exclude the possibility that the feed consumption results in the current study were partially affected by feed wastage, since feed consumption was defined as feed disappearance without adjusting for feed spillage or the amount of feed that may have been eaten by piglets. However, the feeders were inspected several times a day and significant feed spillage was not observed.

### Sow and litter performance

There were no significant differences in average sow BW between the treatment groups throughout the experiment. On average, the sows that received the contaminated diets had a higher weight loss during lactation than the control sows, but the difference was not statistically significant. The lack of significant effect of DON on sow BW changes in the current study concurs with Friends et al. [4], Jakovac-Strajn et al. [9], and Gutzwiller [5]. They found that feeding gestating and lactating sows with diets containing higher DON levels (2.8 to 6.2 mg/kg) had no significant effect on BW changes. However, our findings differ from Chavez [6], who found a significant reduction in BW gain in sows fed a diet containing 3.3 mg/DON kg during gestation, and significantly greater BW losses in sows fed diets containing 1.3 and 2.4 mg DON/kg than controls during lactation. Diaz-Llano and Smith [7, 17] also reported that sows fed diets containing 5.5 mg DON/ kg showed a reduction in BW gain during the last 3 weeks of gestation and greater BW losses during lactation. In another experiment by Diaz-Llano et al. [8], feeding diets containing 3.6 mg DON/kg to sows during gestation and lactation resulted in significantly greater BW losses compared to controls during lactation, while BW gain was not affected during late gestation. Our findings differ also from Herkelman et al. [10], who reported a significant reduction in sow weaning weight and significant higher BW losses in sows fed diets containing 3 mg DON/kg during lactation.

Some previous studies have shown that a high concentration of DON (6.2 mg/kg) exhibited no effect on BW [4], while a lower level of DON (3 to 3.6 mg/kg) reduced BW in the lactating sows [8, 10]. This variability in results may be due to the presence of other mycotoxins in Diaz-Llano et al. [8]. However, in Friend et al. [4] and in the current study, increased DON concentrations were the dominant mycotoxin contamination recorded in the experimental feeds.

In the current study, the backfat thickness of the sows during lactation was measured as an additional indicator of feed efficiency and body condition [19, 20]. We are only aware of one previous study that measured the effect of DON on sow backfat [10]. Our results showed that diets with 1.4 and 1.7 mg DON/kg did not have a statistically significant effect on backfat thickness, although the average losses in backfat thickness in the sows fed contaminated feeds were somewhat higher than in the controls. These results are in accordance with our findings on BW changes in sows. Our findings are in contrast with the results from Herkelman et al. [10], who found significant higher backfat losses in sows fed diets containing 3 mg DON/kg during lactation.

The dietary treatments in the present study did not have significant effect on litter performance. This finding indicates that milk production of the sows was not affected by the treatment diets. This concurs with similar previous studies [4–7, 10, 17]. However, Jakovac-Strajn et al. [9] showed that litter weight gain in the group receiving 5 mg DON/kg was significantly lower than the control. In the same experiment, farrowing was significantly longer in sows receiving 5 mg DON/kg than in the control sows.

The positive correlation between total weight loss during lactation and litter weaning weight, regardless of treatment and parity, was also reported by Thingnes et al. [21]. In the case of feeding sows with DON-contaminated diets, the positive correlation between these two parameters may have contributed to a reduction in the statistical significance of the DON-related effects. In lactation, there is a priority to produce milk over maintenance of sow body tissues [22], but there is considerable individual variation. The reduction in ADFI that was induced by DON-contaminated feed in this study may have led to a reduction in BW in some of the sows, but affected milk production and litter performance more strongly in others. Taken together, this may have reduced the effect of the DON-contaminated feed on both parameters, statistically.

A disturbance of the normal weaning-to-service interval has a considerable negative impact on results in modern piglet production units, because of the use of group farrowing. In the current study, DON-contaminated diets had no effect on the subsequent number of sows that returned to heat at 5 days post-weaning. This finding is in line with the limited, non-significant effect of DON-contaminated diets on BW loss during lactation. Gutzwiller [5] also found no effect of a diet containing 2.8 mg DON/kg on the weaning-to-service interval. However, Diaz-Llano and Smith [7] reported that sows fed contaminated diets with 5.5 mg DON/kg had a trend (*p* = 0.09) toward a longer weaning-to-service interval than the controls, in line with the higher BW losses observed in that experiment.

### Skin temperature

We recorded sow skin temperature as an indicator of the clinical and physiological effects on the animals. Soerensen and Pedersen [23] reviewed using skin temperature for monitoring pig health. In the present study, the sows that received the DON level 2 diet had significantly lower skin temperature than the control group immediately after farrowing. Additionally, their temperature was significantly lower than the DON level 1 group at weaning. However, for all the other skin temperature recordings, no differences between the feeding groups were observed. We cannot exclude the possibility that the significant differences that we found immediately after farrowing and at weaning were accidental. However, sows might have been more sensitive to the effects of DON on skin temperature around farrowing than during the lactation [24]. In some previous studies, feeding weaning pigs with diets containing 3 mg DON/kg resulted in lower skin temperature than in the control pigs [25, 26].

### Hematological and biochemical parameters

There are few reports concerning the hematological and biochemical effects of DON-contaminated diets on gestating and lactating sows. However, DON-related changes in blood parameters have been studied more thoroughly in growing pigs [2, 13]. Our study did not detect any effects of DON on the evaluated hematological and biochemical parameters. Our results concurred with Diaz-Llano and Smith [17], who fed gilts in late gestation with 5.5 mg DON/kg. In contrast, serum urea concentration was reduced in sows that were fed diets with 3.6 and 5.5 mg DON/kg during late gestation and lactation [7, 8].

## Conclusion

In summary, we observed reduced ADFI during lactation in sows that were fed naturally contaminated diets (up to 1.7 mg DON/kg). However, this level of contamination did not have statistically significant effects on sow BW changes during gestation and lactation. Likewise, there were neither effects on production or reproduction performance, nor on blood parameters of the sows.

The highest level of DON contamination in the feed that was used in our study was lower than in most of the previous studies. Nevertheless, it was about twice that of the recommended maximum acceptable level for DON (0.9 mg/kg), according to European Commission Recommendation 2006/576/EC, and more than three times higher than the maximum level (0.5 mg DON/kg) recommended by the Norwegian Food Safety Authority. One of our objectives was to investigate whether sows in a modern, commercial, high-yield piglet production unit might be more sensitive to the effects of DON-contaminated feed than previously documented under other experimental conditions. Our results do not give any indication in that direction. On the contrary, the effects of naturally contaminated feed with 1.4 and 1.7 mg DON/kg were not stronger than the effects previously observed in fattening pigs that were fed similar DON levels [2]. Whether moderate DON levels may have other, more subtle effects on sow health and performance in the long run, is a natural question for further research.

The current study was conducted in a SPF piglet production unit. The sows’ health was particularly good, since they were free from many common pathogens. The SPF status of our study herd might have enhanced sow resistance to DON-related toxicity. This issue may also require future study.

## Additional file

Additional file 1: **Table S1.** Toxin contents in the oats used for the production of the experimental diets, as measured by multi-toxin LC-MS/MS by the Centre for Analytical Chemistry at IFA Tulln, Austria. (DOCX 34 kb)

## Abbreviations

ADFI: Average daily feed intake
ALP: Alkaline phosphatase
ANOVA: Analysis of variance
AST: Aspartate aminotransferase
BCS: Body condition scores
BW: Body weight
CK: Creatine kinase
CRP: C-reactive protein
DON: Deoxynivalenol
EDTA: Ethylenediaminetetraacetic acid
GGT: Gamma-glutamyl transpeptidase
GLDH: Glutamate dehydrogenase
HSD: Honest significant difference
NMBU: Norwegian University of Life Sciences
SD: Standard deviation
SPF: Specific pathogen-free

## References

[1] Goyarts T, Danicke S (2006). Bioavailability of the Fusarium toxin deoxynivalenol (DON) from naturally contaminated wheat for the pig. Toxicol Lett.

[2] Bergsjø B, Langseth W, Nafstad I, Jansen JH, Larsen HJ (1993). The effects of naturally deoxynivalenol-contaminated oats on the clinical condition, blood parameters, performance and carcass composition of growing pigs. Vet Res Commun.

[3] Eriksen GS, Pettersson H (2004). Toxicological evaluation of trichothecenes in animal feed. Anim Feed Sci Technol.

[4] Friend DW, Thompson BK, Trenholm HL, Hartin KE, Prelusky DB (1986). Effects of feeding deoxynivalenol (DON)-contaminated wheat diets to pregnant and lactating gilts and on their progeny. Can J Anim Sci.

[5] Gutzwiller A (2010). Effects of deoxynivalenol (DON) in the lactation diet on the feed intake and fertility of sows. Mycotoxin Res.

[6] Chavez ER (1984). Vomitoxin-contaminated wheat in pig diets: pregnant and lactating gilts and weaners. Can J Anim Sci.

[7] Diaz-Llano G, Smith TK (2007). The effects of feeding grains naturally contaminated with Fusarium mycotoxins with and without a polymeric glucomannan adsorbent on lactation, serum chemistry, and reproductive performance after weaning of first-parity lactating sows. J Anim Sci.

[8] Diaz-Llano G, Smith TK, Boermans HJ, Caballero-Cortes C, Friendship R (2010). Effects of feeding diets naturally contaminated with Fusarium mycotoxins on protein metabolism in late gestation and lactation of first-parity sows. J Anim Sci.

[9] Jakovac-Strajn B, Vengust A, Pestevsek U (2009). Effects of a deoxynivalenol-contaminated diet on the reproductive performance and immunoglobulin concentrations in pigs. Vet Rec.

[10] Herkelman KL, Hall RE, Martel-Kennes Y (2017). Influence of diet contaminated with deoxynivalenol (DON) on lactating sow and litter performance. J Anim Sci.

[11] Mysen E (2011). Mykotoksiner ga problemer (Mycotoxins gave problems). Svin.

[12] Thingnes SL. The impact of diet and feeding strategies on gilt and sow performance [dissertation]. Oslo: Norwegian university of life sciences; 2013.

[13] Sayyari A, Fæste CK, Hansen U, Uhlig S, Framstad T, Schatzmayr D, Sivertsen T (2018). Effects and biotransformation of the mycotoxin deoxynivalenol in growing pigs fed with naturally contaminated pelleted grains with and without the addition of Coriobacteriaceum DSM 11798. Food Addit Contam Part A.

[14] Malachova A, Sulyok M, Beltran E, Berthiller F, Krska R (2014). Optimization and validation of a quantitative liquid chromatography-tandem mass spectrometric method covering 295 bacterial and fungal metabolites including all regulated mycotoxins in four model food matrices. J Chromatogr A.

[15] Ivanova L, Sahlstrøm S, Rud I, Uhlig S, Fæste CK, Eriksen GS, Divon HH (2017). Effect of primary processing on the distribution of free and modified Fusarium mycotoxins in naturally contaminated oats. World Mycotoxin J.

[16] Framstad T, Sjaastad Ø, Aass RA (1988). Blodprøvetaking på gris (Blood sampling in pigs). Nor Vet Tidsskr.

[17] Diaz-Llano G, Smith TK (2006). Effects of feeding grains naturally contaminated with Fusarium mycotoxins with and without a polymeric glucomannan mycotoxin adsorbent on reproductive performance and serum chemistry of pregnant gilts. J Anim Sci.

[18] Øvernes G, Matre T, Sivertsen T, Larsen HJ, Langseth W, Reitan LJ, Jansen JH (1997). Effects of diets with graded levels of naturally deoxynivalenol-contaminated oats on immune response in growing pigs. Zentralbl Veterinarmed A.

[19] Maes DGD, Janssens GPJ, Delputte P, Lammertyn A, de Kruif A (2004). Back fat measurements in sows from three commercial pig herds: relationship with reproductive efficiency and correlation with visual body condition scores. Livest Prod.

[20] Theil PK, Lauridsen C, Quesnel H (2014). Neonatal piglet survival: impact of sow nutrition around parturition on fetal glycogen deposition and production and composition of colostrum and transient milk. Anim: Int J Anim Biosci.

[21] Thingnes SL, Gaustad AH, Kjos NP, Hetland H, Framstad T (2013). Pea starch meal as a substitute for cereal grain in diets for lactating sows: the effect on sow and litter performance. Livest Sci.

[22] Close WH, Cole DJA. Nutrition of sows and boars. Nottingham: Nottingham University Press; 2000.

[23] Soerensen DD, Pedersen LJ (2015). Infrared skin temperature measurements for monitoring health in pigs: a review. Acta Vet Scand.

[24] Terøy MM, Helland EM, Framstad T (2014). Use of infrared technology to detect rise in surface temperature in sows at farrowing. European Symposium of Porcine Health Management (ESPHM).

[25] Prelusky DB, Gerdes RG, Underhill KL, Rotter BA, Jui PY, Trenholm HL (1994). Effects of low-level dietary deoxynivalenol on haematological and clinical parameters of the pig. Nat Toxins.

[26] Rotter BA, Thompson BK, Lessard M, Trenholm HL, Tryphonas H (1994). Influence of low-level exposure to Fusarium mycotoxins on selected immunological and hematological parameters in young swine. Fundam App Toxicol: Off J Soc Toxicol.

