# Supplementary material for: Effects of feeding naturally contaminated deoxynivalenol diets to sows during late gestation and lactation in a high-yield specific pathogen-free herd
Source: Porcine Health Manag. 2018 Nov 1;4:26. doi: 10.1186/s40813-018-0102-9 (PMC6211461; doi:10.1186/s40813-018-0102-9)
Supplement: Supplementary file 1 — Table S1. Toxin contents in the oats used for the production of the experimental diets, as measured by multi-toxin LC-MS/MS by the Centre for Analytical Chemistry at IFA Tulln, Austria. (DOCX 34 kb) [file 40813_2018_102_MOESM1_ESM.docx]

Additional file 1: **Table S1** Toxin contents in the oats used for the production of the experimental diets, as measured by multi-toxin LC-MS/MS by the Centre for Analytical Chemistry at IFA Tulln, Austria.

|  | **Oats ^a^** | | |
| --- | --- | --- | --- |
| **Toxins (µg/kg)** | **Sample 1** |  | **Sample 2** |
| 15-Hydroxyculmorin | 499 |  | 500 |
| 15-Hydroxyculmoron | 182 |  | 193 |
| 5-Hydroxyculmorin | 387 |  | 441 |
| Alternariol | 15.3 |  | 2.12 |
| Alternariolmethylether | 1.58 |  | 0.64 |
| Andrastin A | < LOD |  | < LOD |
| Apicidin | 54.5 |  | 49.5 |
| Ascochlorin | 6.37 |  | 0.98 |
| Asperfuran | < LOD |  | < LOD |
| Asperglaucide | 5.38 |  | 3.91 |
| Asperphenamate | 12.08 |  | 9.51 |
| Aspterric acid | < LOD |  | < LOD |
| Aurofusarin | 1771 |  | 1310 |
| Beauvericin | 13.3 |  | 14.4 |
| Brevianamid F | 10.4 |  | 9.15 |
| Butenolid | 19.6 |  | 21.9 |
| Chanoclavin | 0.24 |  | 0.27 |
| Chrysogin | 134 |  | 144 |
| Citreorosein | 5.42 |  | 5.83 |
| Culmorin | 2139 |  | 1996 |
| Curvularin | 14.4 |  | 7.52 |
| cyclo(L-Pro-L-Tyr) | 79.2 |  | 63.9 |
| cyclo(L-Pro-L-Val) | 95.5 |  | 75.9 |
| Deoxynivalenol | 3788 |  | 3975 |
| Diplodiatoxin | < LOD |  | < LOD |
| DON-3-glucoside | 843 |  | 869 |
| Elymoclavine | < LOD |  | < LOD |
| Emodin | 4.86 |  | 5.70 |
| Enniatin A | 0.39 |  | 0.36 |
| Enniatin A1 | 2.83 |  | 3.09 |
| Enniatin B | 14.9 |  | 14.7 |
| Enniatin B1 | 13.0 |  | 13.2 |
| Enniatin B2 | 0.69 |  | 0.81 |
| Enniatin B3 | 0.00 |  | 0.00 |
| Epiequisetin | 0.85 |  | 0.91 |
| Equisetin | 1.88 |  | 1.16 |
| Ergometrine | < LOD |  | 10.44 |
| Ergometrinine | 0.35 |  | 0.82 |
| Fallacinol | < LOD |  | < LOD |
| Fellutanine A | 15.5 |  | 11.9 |
| Fonsecin | < LOD |  | < LOD |
| Fumonisin B1 | < LOD |  | < LOD |
| Fumonisin B2 | < LOD |  | < LOD |
| Fumonisin B3 | < LOD |  | < LOD |
| Fusaric acid | < LOD |  | < LOD |
| Fusarinolic acid | < LOQ ^b^ |  | < LOQ |
| HT-2 toxin | 9.82 |  | < LOD |
| Infectopyron | 175 |  | 189 |
| Iso-Rhodoptilometrin | < LOD |  | 0.30 |
| Kojic acid | < LOD |  | < LOD |
| Macrosporin | < LOD |  | < LOD |
| Moniliformin | 9.46 |  | 28.3 |
| Mycophenolic acid | < LOD |  | < LOD |
| N-Benzoyl-Phenylalanine | 5.07 |  | 3.48 |
| Neoechinulin A | 22.6 |  | 33.1 |
| Nivalenol | 402 |  | 399 |
| Ochratoxin A | < LOD |  | < LOD |
| Pestalotin | < LOD |  | < LOD |
| Physcion | < LOD |  | < LOD |
| Questiomycin A | < LOD |  | < LOD |
| Quinolactacin A | 0.14 |  | 0.10 |
| Rugulusovin | 5.00 |  | 3.58 |
| Siccanol | < LOQ |  | < LOQ |
| T-2 toxin | 10.1 |  | < LOD |
| Tentoxin | 1.24 |  | 2.08 |
| Tenuazonic acid | 82.6 |  | 40.2 |
| Tryptophol | 111 |  | 85.3 |
| Xanthotoxin | < LOD |  | < LOD |
| Zearalenone | 38.1 |  | 58.5 |

^a^ The samples 1 and 2 are the replicates of the same batch of oats.

^b^ Limit of Quantitation
